# Royal jelly attenuates metabolic defects in a *Drosophila* mutant with elevated TORC1 activity

**DOI:** 10.1242/bio.054999

**Published:** 2020-11-06

**Authors:** Yang Cheng, Jiadong Cai, Yuanyuan Fu, Congjing Feng, Yue Hao, Youheng Wei

**Affiliations:** 1 College of Bioscience and Biotechnology, Yangzhou University, Yangzhou 225009, China; 2 Joint International Research Laboratory of Agriculture and Agri-Product Safety, the Ministry of Education of China, Yangzhou University, Yangzhou 225009, China; 3 Institute of Metabolism and Reproduction, Yangzhou University, Yangzhou 225009, China; 4 College of Horticulture and Plant Protection, Yangzhou University, Yangzhou 225009, China; 5 Key Laboratory of Pollinating Insect Biology, Ministry of Agriculture, Beijing 100093, China

**Keywords:** *Drosophila melanogaster*, Royal jelly, Target of rapamycin complex 1, Antioxidant activity, Metabolism regulation

## Abstract

Target of rapamycin complex 1 (TORC1) is a master regulator of cell metabolism, and its dysregulation has been linked to an array of pathologies, including cancer and age-related diseases. Nprl3, a component of GTPase-activating protein towards Rags complex 1 (GATOR1), inhibits TORC1 activity under nutrient scarcity status. The *nprl3* mutant exhibits some metabolic defects due to hyper TORC1 activity in *Drosophila*. Royal jelly (RJ) is a honeybee-secreted product and plays an essential role in caste differentiation that requires TORC1 activity. RJ is also used as a health-benefit food for its potential roles on antioxidant and anti-aging. In this study, *nprl3*-mutant flies were used to measure the effect of RJ on metabolic modulation. Interestingly, RJ feeding significantly increased survival and decreased TORC1 activity in the *nprl3* mutant. RJ feeding also ameliorated the abnormal reactive oxygen species (ROS) levels and energy status in the *nprl3* mutant. The proteins in RJ were characterized to be the essential components in increasing *nprl3* mutant viability. These findings suggest that RJ modulates some metabolic defects associated with elevated TORC1 activity and that the *nprl3*-mutant fly might be a useful tool for investigating the bioactive components of RJ *in vivo*.

## Abstract (precis)

**Summary:** Royal jelly (RJ) modulates the reactive oxygen species (ROS) levels and some metabolic defects that associated with hyper TORC1 activity in a *Drosophila* model.

## INTRODUCTION

Target of rapamycin complex 1 (TORC1) is a master regulator that mediates nutrient status and metabolism. It promotes protein synthesis and cell growth by phosphorylating downstream effectors, such as p70 S6 kinase (S6K) and eIF-4E binding protein (4E-BP) (Bar-Peled and Sabatini, 2014; Kim and Guan, 2019). The inhibition of TORC1 increases the longevity of a variety of model organisms, whereas the elevated TORC1 activity has been associated with a variety of age-related diseases, including cancer, diabetes, and Parkinson's (Johnson et al., 2013; Laplante and Sabatini, 2012; Zoncu et al., 2011). Nprl3 is a component of GTPase-activating protein towards Rags complex 1 (GATOR1), which inhibits TORC1 activity under nutrient starvation (Bar-Peled et al., 2013; Wei and Lilly, 2014; Wei et al., 2014). The *nprl3*-mutant flies exhibit increased TORC1 activity and a variety of related phenotypes, such as semi-lethality, short lifespan, decreased motility, and some metabolic defects (Wei et al., 2016).

Multiple studies showed that high TORC1 activity promotes the generation of reactive oxygen species (ROS) (Chen et al., 2008; Kim et al., 2005). ROS are byproducts of aerobic metabolism during which the oxygen gets one electron and forms free radical superoxide including superoxide anion (O_2_^−^), hydrogen peroxide (H_2_O_2_), and hydroxyl radical (HO•) (Zorov et al., 2014). ROS can react and cause oxidative damage to macromolecules like protein, DNA and lipid, and thus are highly toxic for cells (Schieber and Chandel, 2014). In physiological conditions, ROS are removed by the antioxidant enzymes such as superoxide dismutases (SOD) and catalase (CAT). Thus, following either an increase in ROS generation or a decrease in antioxidant capacity, oxidative stress occurs and causes pathologies such as aging and age-related diseases (Schieber and Chandel, 2014).

Royal jelly (RJ), a nutrient-rich liquid, is secreted by the hypopharyngeal and mandibular glands of young nurse honeybees (*Apis mellifera*) (Ramadan and Al-Ghamdi, 2012). In *Apis mellifera*, RJ is an essential component for larvae differentiation to workers or queens that have the same genetic background (Colhoun and Smith, 1960; Maleszka, 2008). A few female larvae fed with RJ throughout the whole larval stage develop into large-bodied, long-lived, fertile queens, whereas most other female larvae provided RJ for the first 3 days after hatching and then switched to the beebread that contains predominantly honey and pollen develop into small-bodied, short-lived, sterile workers (Colhoun and Smith, 1960; Page and Peng, 2001). Recently, TORC1 activity is taken as an essential factor for caste differentiation in *Apis mellifera*. Both pharmacological treatment and genetic manipulation that decreased *Apis mellifera* TOR (amTOR) activity block queens’ fates (Mutti et al., 2011; Patel et al., 2007; Wheeler et al., 2014; Zhu et al., 2017). Furthermore, RJ and the RJ protein, royalactin, promote the differentiation to queens through EGFR pathway with increased S6K activity, which is also a downstream effector of TORC1 (Kamakura, 2011). These findings suggest that TORC1 activity is required for caste differentiation of larvae into queens.

One interesting question is whether RJ supplemental food promotes TORC1 activity in other animals with similar effects to those seen in *Apis mellifera*. If it was true, the RJ food might have an adversarial effect on health, especially for individuals with hyper-TORC1 activity, which is closely associated with aging and aging-related diseases. RJ has been traditionally used in some countries as food supplement and been reported to have health benefits, including antioxidant, antimicrobial, anti-inflammatory, anti-tumor, and anti-aging properties (Fratini et al., 2016; Pasupuleti et al., 2017; Ramadan and Al-Ghamdi, 2012). However, RJ is not allowed to be sold as a health-beneficial food for humans in Europe or the USA (Shorter et al., 2015).

Accordingly, the present study uses *nprl3*-mutant flies to investigate the effect of RJ on animals with hyper TORC1 activity. Interestingly, RJ feeding significantly alleviated the hyper TORC1 activity and increased the viability, antioxidase activity, and energy levels in *nprl3* flies. Additionally, the proteins in RJ were found to be essential components for its function. These results suggest that RJ attenuates the metabolic defects associated with hyper TORC1 levels and provides a model system for quickly screening the bioactive components of RJ.

## RESULTS

### RJ improves the survival of *nprl3*-mutant flies

**Fig. 1.**
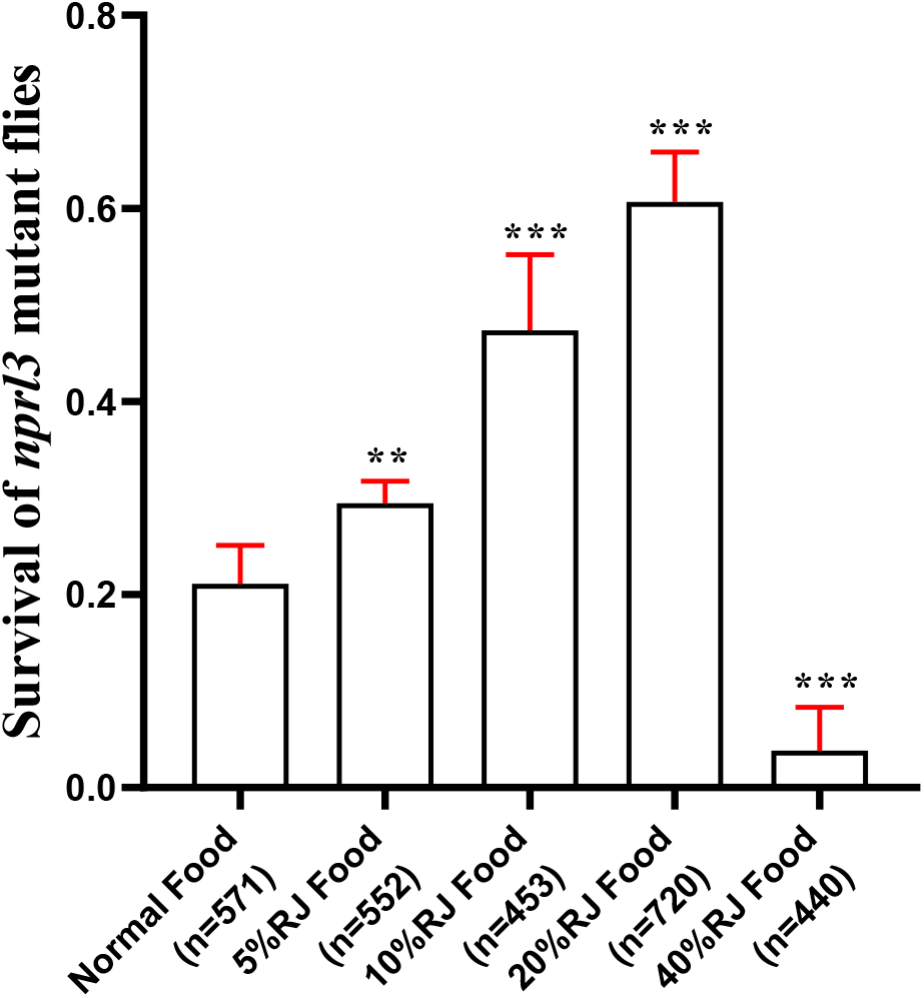
**Effect of dietary royal jelly (RJ) on the viability of *nprl3*-mutant flies.** The viability ratio of *nprl3*-mutant flies on different foods was calculated; n, number of total hatched adult flies; error bars indicate s.d. of five independent repeats; **P<0.01; ***P<0.001 compared with normal food group.

The *nprl3*-mutant flies are semi-lethal and display multiple age-related pathologies and metabolic defects, owing to elevated TORC1 activity (Wei et al., 2016). RJ exhibits a variety of beneficial properties, including metabolism regulation and antioxidant properties in multiple organisms (Ramadan and Al-Ghamdi, 2012). To determine the effect of RJ function on modulating metabolism defects with hyper TORC1 activity, the *nprl3*-mutant flies were fed with food containing RJ. Interestingly, RJ-feeding significantly increased the viability of the *nprl3* mutant (Fig. 1). Because 20% RJ had the best effect on increasing the viability of *nprl3* mutants, this concentration was used in the following study.

**Fig. 2.**
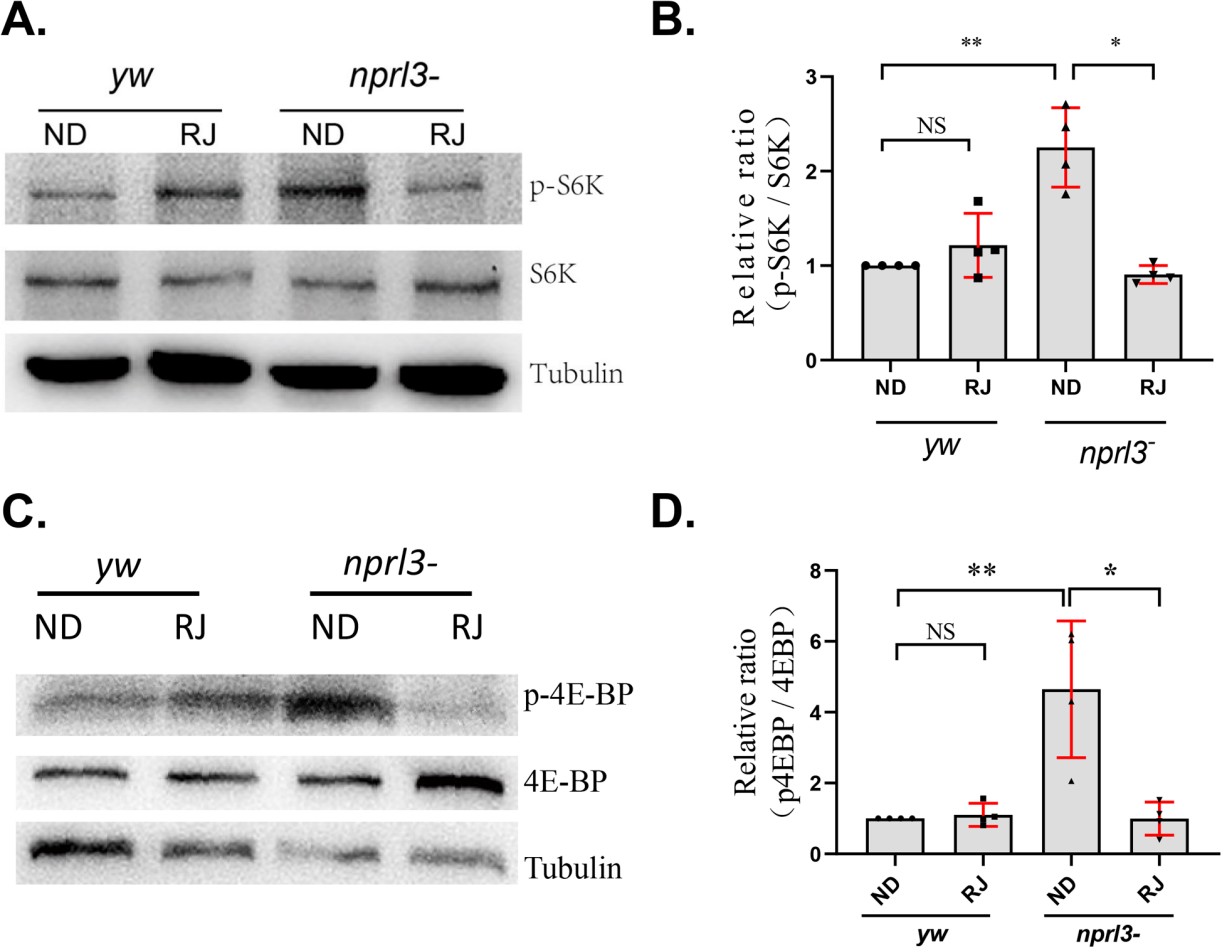
**RJ feeding decreases TORC1 activity in the *nprl3* mutant*.*** *yw* and *nprl3^1^/Df* flies were collected from normal food or RJ food. (A) Phosphorylated S6K (top), total S6K (middle), and Tubulin (bottom) in male flies were detected using western blot. (B) The relative intensity of phosphorylated S6K band to total S6K band. (C) Phosphorylated 4E-BP (top), total 4E-BP (middle), and Tubulin (bottom) in male flies were detected using western blot. (D) The relative intensity of phosphorylated 4E-BP band to total 4E-BP band. The ratio of p4E-BP/4E-BP in the *yw* was set as 1. Data are presented as mean±s.d.; values are from four independent experiments; *P<0.05; **P<0.001; NS, not significant; *yw*, control flies; *nprl3-*, *nprl3^1^/Df* flies; ND, normal diet; RJ, 20% RJ diet.

To investigate the effect of RJ feeding on TORC1 activity, the phosphorylation level of the TORC1 downstream effectors S6K and 4E-BP were determined. Consistent with our previous report (Wei et al., 2016), the TORC1 activity significantly increased in *nprl3*-mutant flies (Fig. 2). Compared with slight but not significant effect on wild-type flies, RJ feeding significantly alleviated the TORC1 activity in *nprl3*-mutant flies (Fig. 2).

### RJ reduces ROS level and enhances oxidase activity in *nprl3*-mutant flies

**Fig. 3.**
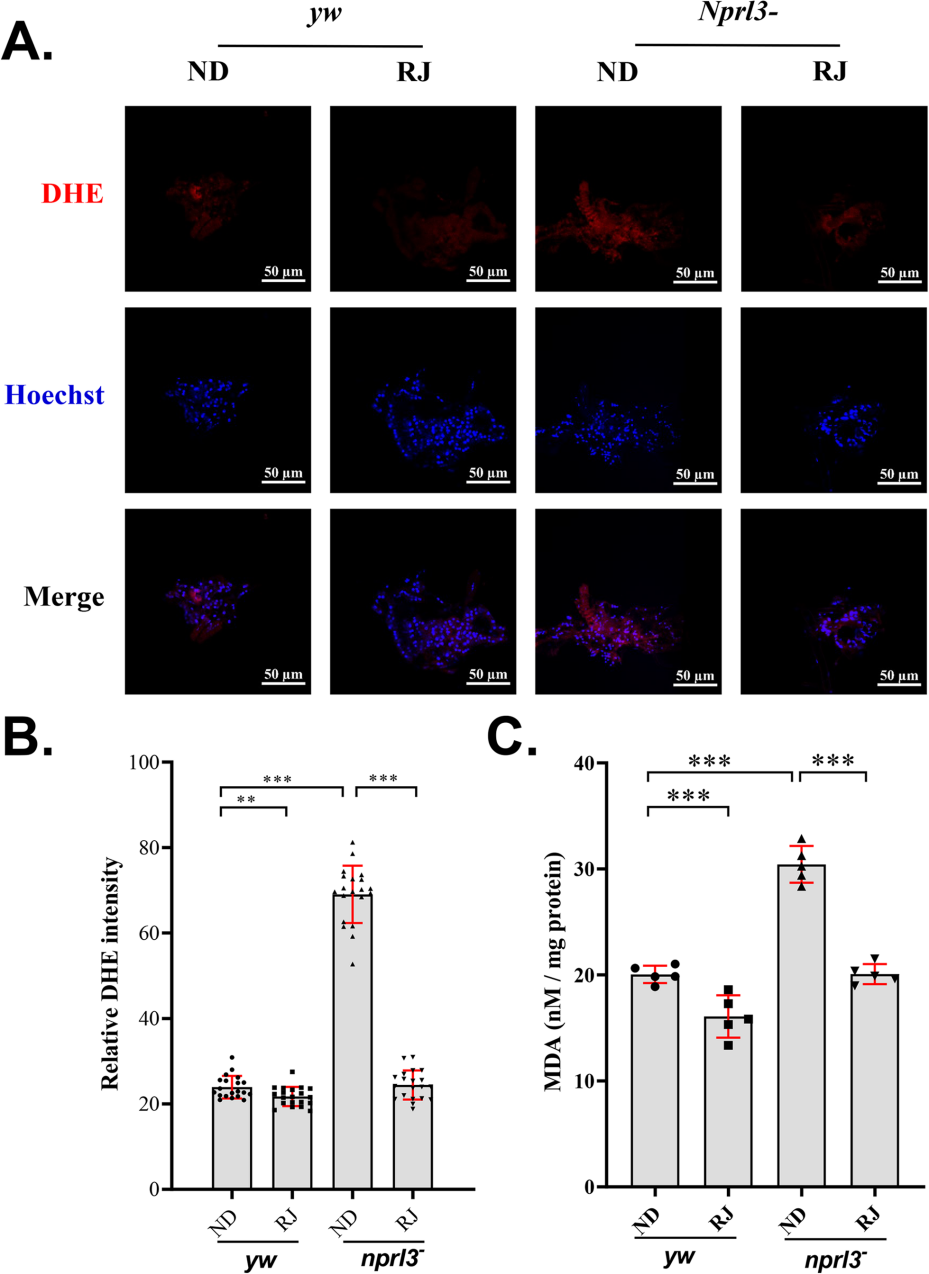
**RJ feeding decreases the reactive oxygen species levels in the *nprl3* mutant*.*** *yw* and *nprl3^1^/Df* flies were collected from normal food or RJ food. (A) DHE (red) and Hoechst (blue) stained fat bodies were imaged using a confocal microscope. (B) The values of DHE intensity were quantified (*n*=20). (C) The MDA levels were determined (*n*=5). Data are presented as mean±s.d. **P<0.01, ***P<0.001. *yw*, control flies, *nprl3^−^*, *nprl3^1^/Df* flies; ND, normal diet; RJ, 20% RJ diet.

The *nprl3* mutant might contain more ROS because TORC1 promotes ROS generation (Chen et al., 2008; Kim et al., 2005). To evaluate the effect of RJ feeding, we detected ROS levels in males, which are comprised primarily of somatic tissues. The Dihydroethidium (DHE) staining method is widely used to detect ROS levels in cells and tissues (Gomes et al., 2005). Interestingly, the DHE staining was stronger in *nprl3*-mutant flies, which can be significantly alleviated by RJ feeding (Fig. 3A,B). In animals, ROS oxidizes unsaturated fatty acids and generates malondialdehyde (MDA), which can be used as an indicator of oxidation (Tsikas, 2017). Consistent with the results of DHE staining, the *nprl3*-mutant flies had more MDA, which was reduced by RJ feeding (Fig. 3C). In addition, RJ feeding slightly reduced the DHE staining and MDA amount in wild-type flies (Fig. 3).

**Fig. 4.**
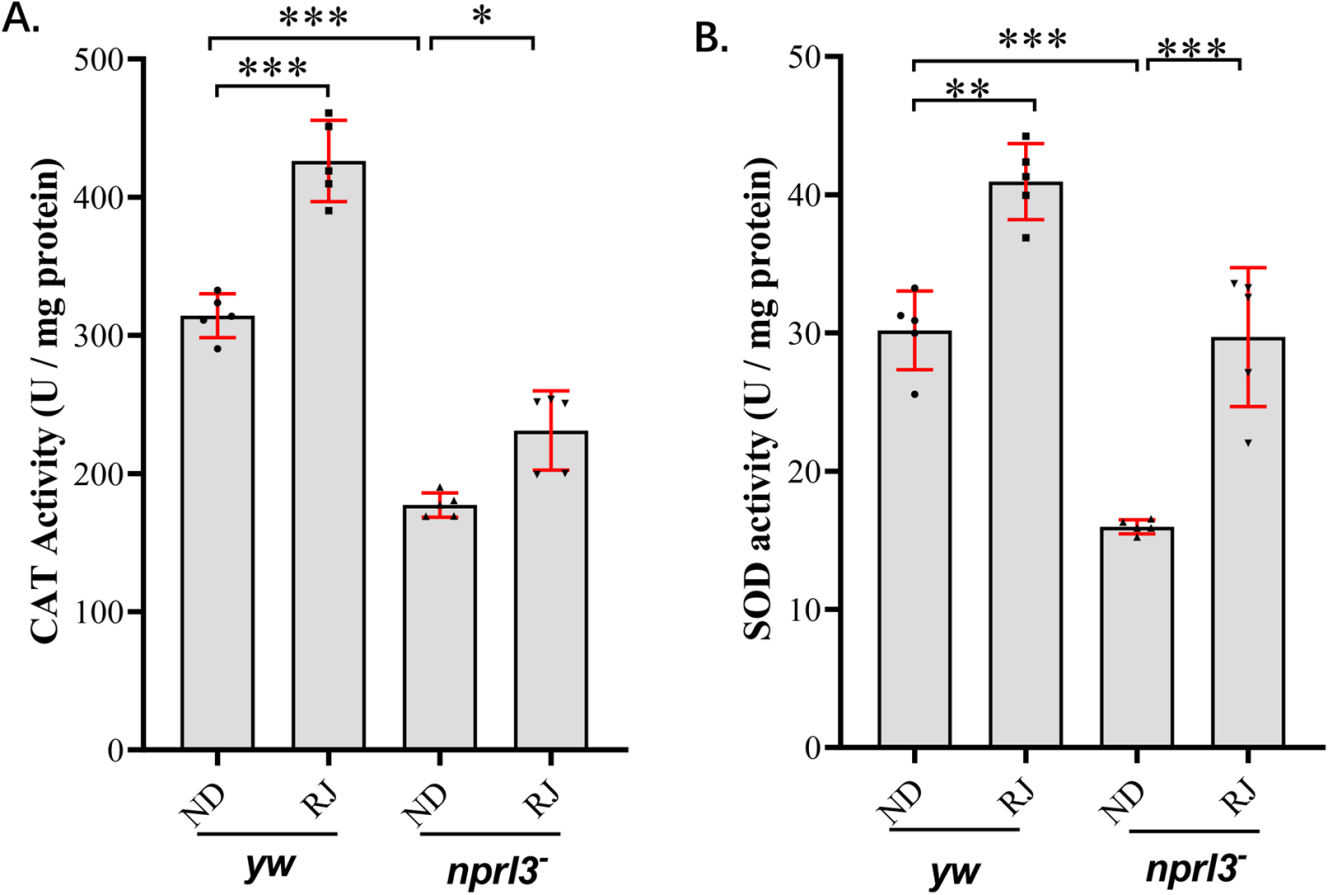
**RJ feeding increases antioxidase activities in the *nprl3* mutant.** *yw* and *nprl3^1^/Df* flies were collected from normal food or RJ food. (A) SOD activities and (B) CAT activities were determined. Data are presented as mean±s.d.; *P<0.05; **P<0.01; ***P<0.001; values are from five independent experiments*;* *yw*, control flies, *nprl3^−^*, *nprl3^1^/Df* flies; ND, normal diet; RJ, 20% RJ diet.

The two canonical antioxidases, SOD and CAT, play important roles in ROS elimination. The SOD and CAT activities of the *nprl3*-mutant flies were significantly lower than those of wild-type flies, which could be rescued by RJ feeding (Fig. 4).

The lethality of *nprl3* mutants occurred at late pupal stage, which might have been caused by the defects at an earlier developmental stage (Wei et al., 2016). To confirm the effect of RJ on reducing ROS levels, we detected the MDA and antioxidase activity at the larval stage. Consistent with the effect in adult flies, RJ feeding improved the antioxidant capacity and decreased the oxidative levels in *nprl3*-mutant larva (Figs S1 and S2).

### RJ improves energy storage and usage in *nprl3*-mutant flies

**Fig. 5.**
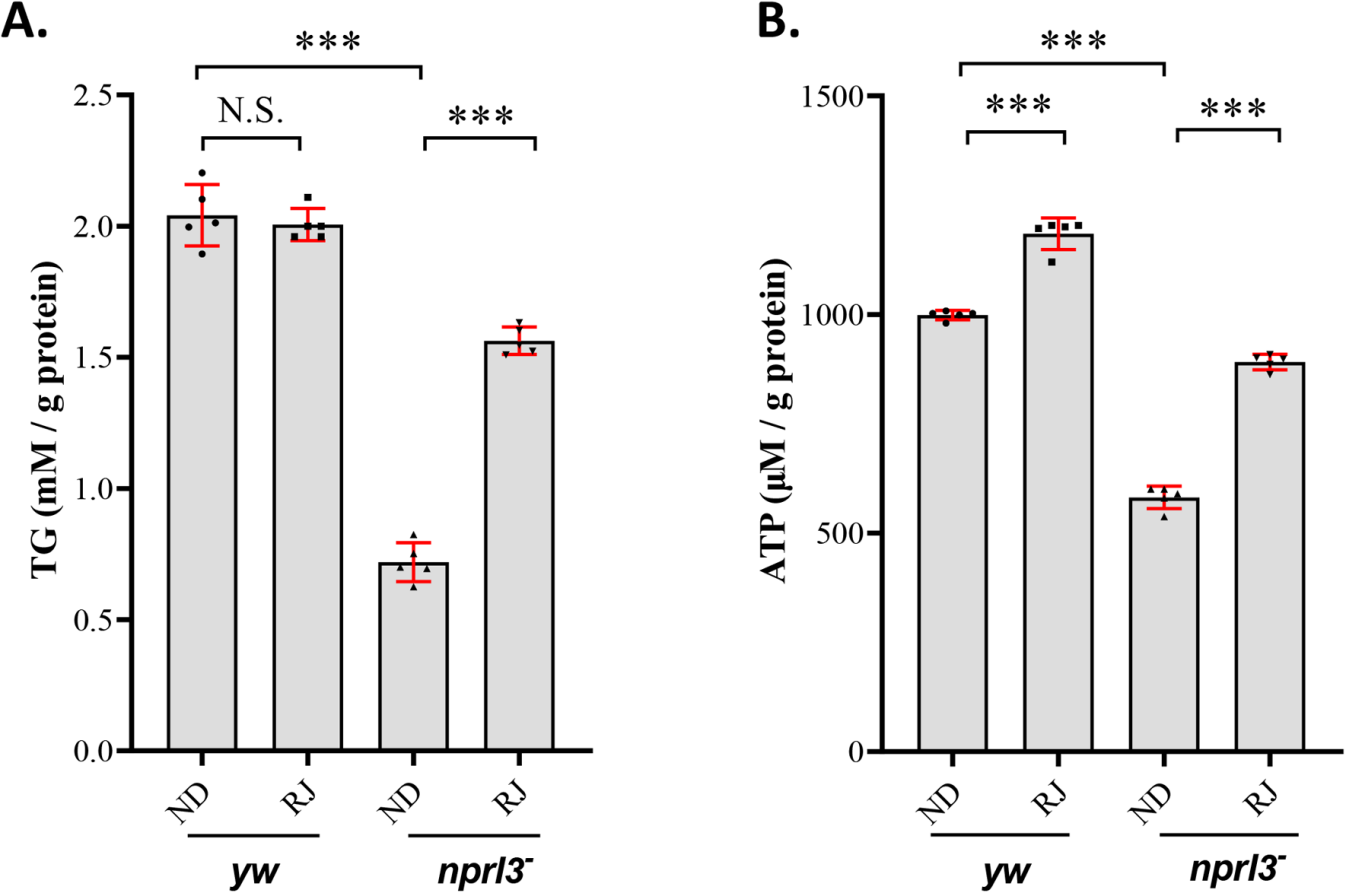
**Effect of RJ on the metabolism in the *nprl3* mutant.** *yw* and *nprl3^1^/Df* flies were collected from normal food or RJ food. (A) Triglyceride levels and (B) ATP levels were determined. Data are presented as mean±s.d; values are from five independent experiments; ***P<0.001; NS, not significant; *yw*, control flies, *nprl3^−^*, *nprl3^1^/Df* flies; ND, normal diet; RJ, 20% RJ diet.

The *nprl3*-mutant flies exhibit reduced triglyceride (TG), which is the most abundant form of stored energy in the *Drosophila* body (Wei et al., 2016). Here we found that RJ feeding significantly increased the TG levels in the *nprl3*-mutant flies (Fig. 5A). In animals, TG is used to produce adenosine triphosphate (ATP), which is considered the energy currency of living cells (Bonora et al., 2012). Indeed, the *nprl3*-mutant flies also possessed lower levels of ATP than the wild-type flies, which could be alleviated by RJ feeding (Fig. 5B). Furthermore, the TG and ATP levels were increased in *nprl3*-mutant larvae, which were alleviated by RJ feeding (Fig. S3). These results suggested that RJ could alleviate the metabolic defects in *nprl3*-mutant flies.

### RJ proteins are essential for modulating the viability of *nprl3* mutants

**Fig. 6.**
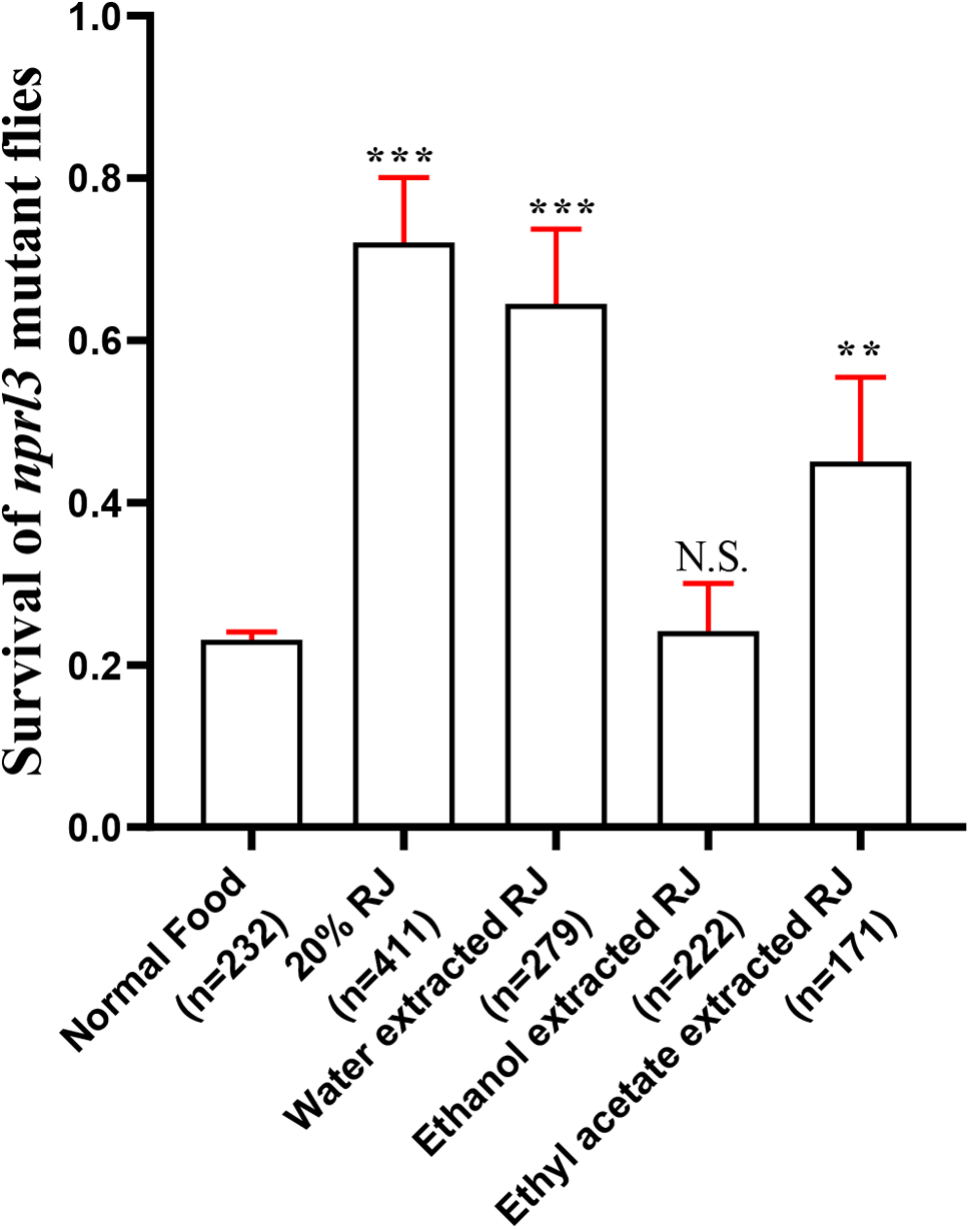
**Effect of isolated RJ on the viability of *nprl3*-mutant flies.** RJ was isolated with water, ethanol or ethyl acetate for making food. The viability ratio of *nprl3*-mutant flies on different food was calculated; n, number of total hatched adult flies; the viability ratio in each repeat is calculated and averaged; error bars indicate s.d. of five independent repeats; **P<0.01; ***P<0.001; NS, not significant compared with normal food group.

Next, we investigated the bioactive components responsible for increasing *nprl3* mutant viability. The effect of RJ components isolated using water, ethanol, or ethyl acetate were detected. The water-isolated RJ components had a similar effect to complete RJ on *nprl3* mutant viability, which suggests that the major bioactive components are water soluble (Fig. 6).

**Fig. 7.**
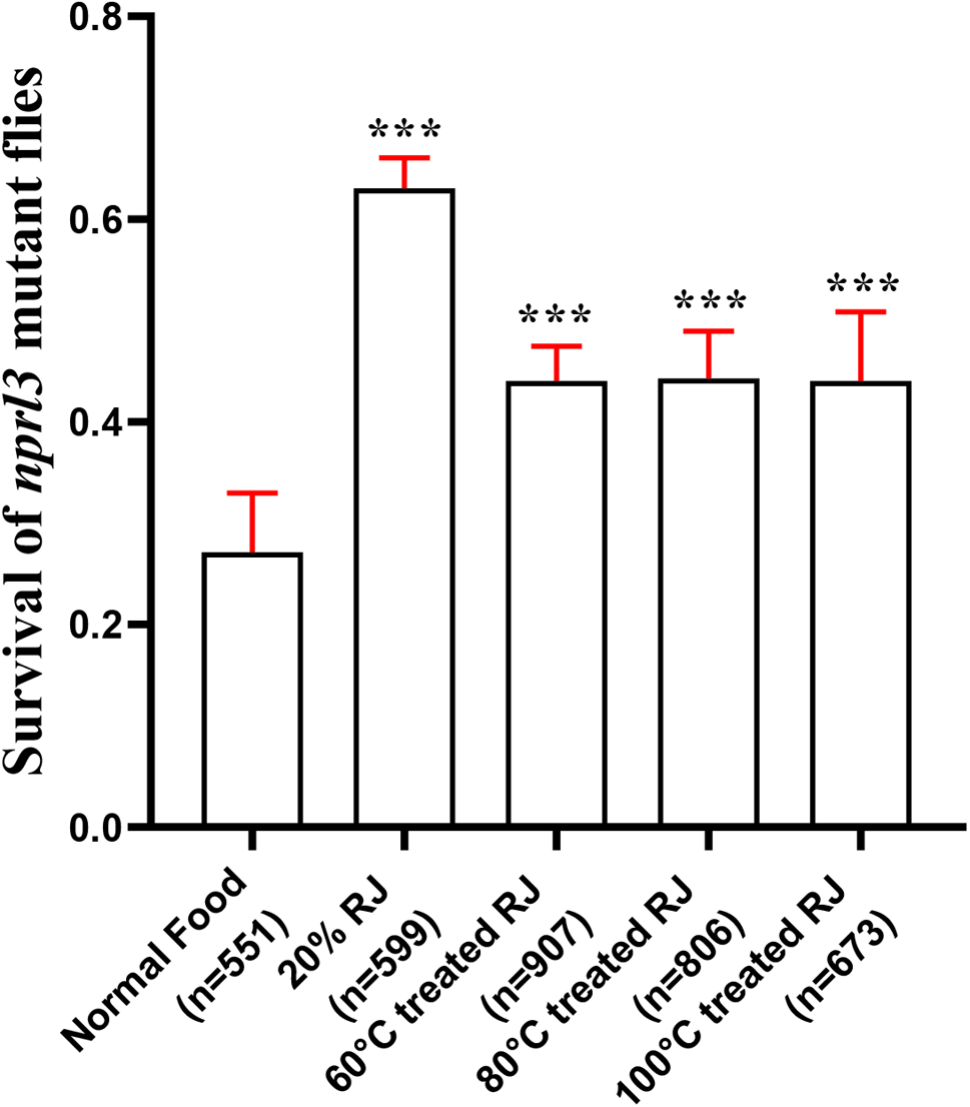
**Effect of high temperature treated RJ on the viability of *nprl3*-mutant flies.** RJ was treated at different temperatures for 30 min and used for making food. The viability ratio of *nprl3*-mutant flies on different foods was calculated. n, number of total hatched adult flies; error bars indicate s.d. of six independent repeats; ***P<0.001 compared with normal food group.

To further identify the character of the bioactive components, RJ was treated at different temperatures before being added to food. Interestingly, the function of RJ on *nprl3* viability significantly decreased when treated at 60°C for 30 min, but did not further decrease at 100°C (Fig. 7). This result suggested that some RJ bioactive components are temperature sensitive.

**Fig. 8.**
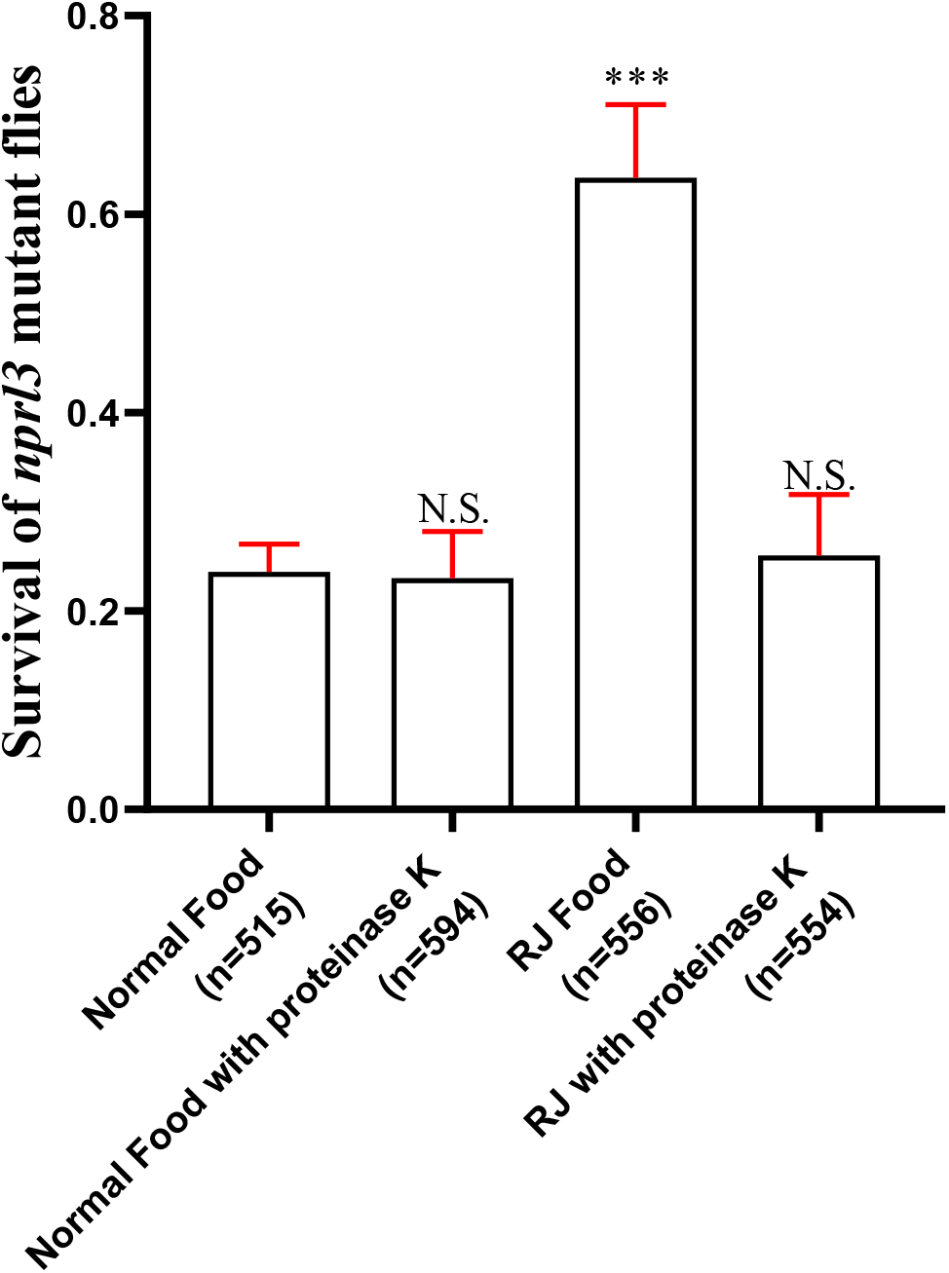
**Effect of proteinase K digested RJ on the viability of *nprl3* mutant flies.** RJ was digested with proteinase K and used for making food. The viability ratio of *nprl3*-mutant flies on different food was calculated. n, number of total hatched adult flies; error bars indicate s.d. of at least four independent repeats; ***P<0.001; NS, not significant compared with normal food group.

Most proteins are water soluble and temperature sensitive. To determine whether proteins are the bioactive component responsible for rescuing *nprl3* mutants, the water-isolated RJ component was treated with proteinase K before being made into food. Interestingly, the proteinase K-digested RJ failed to improve the viability of the *nprl3*-mutant flies (Fig. 8), which suggested that RJ proteins are essential components for rescuing the metabolic defects of *nprl3*-mutant flies.

## DISCUSSION

TORC1 promotes anabolic processes and stimulates oxygen consumption and mitochondrial capacity to provide energy for protein synthesis (Ramanathan and Schreiber, 2009). During mitochondrial respiration, ROS is generated as a byproduct, especially in damaged mitochondria (Zorov et al., 2014). In addition, TORC1 inhibits autophagy, the process of eliminating the damaged mitochondria. Here, we found that mutation of *nprl3,* a component of the TORC1 inhibitor GATOR1, results in a high ROS level in *Drosophila*. ROS stress causes accumulated organelle damage and accelerates aging in traditional views (Liochev, 2013). The GATOR1-mutant flies, including *nprl2*, *nprl3* and *iml1*, display multiple age-related defects that might be associated with high ROS levels (Wei et al., 2016; Xi et al., 2019). Furthermore, we observed low SOD and CAT activities in *nprl3* mutants, which suggests that hyper TORC1 inhibits the elimination of ROS. This result is consistent with the report that TORC1 phosphorylates and inhibits SOD1 activity in yeast and human cells (Tsang et al., 2018). The RJ food significantly alleviates the hyper TORC1 activity in *nprl3*-mutant flies, and this might be related to its effect on ROS clearance. Our results suggest that RJ feeding alleviates the metabolic defects of the animals with hyper TORC1 activity. The RJ exerted a similar tendency on modulating ROS and metabolism between wild-type and *nprl3*-mutant flies at both larval and adult stages. Furthermore, that RJ feeding increased the viability of *nprl3*-mutant flies might be related to its effect at the larval stage. Thus, the *nprl3* mutant might be a useful tool for identifying RJ bioactive materials.

We found that some water-soluble, temperature-sensitive or -resistant RJ proteins are essential components for RJ biological function. Previously, the RJ proteins have been reported to function on modulating metabolism (Fratini et al., 2016; Guo et al., 2008; Barnutiu et al., 2011; Ramadan and Al-Ghamdi, 2012). Some RJ proteins, such as the oligo form major royal jelly protein 1 (MRJP1) are temperature resistant, while the mono form MRJP1 is temperature sensitive (Moriyama et al., 2015). Further study is needed to identify the specific proteins and evaluate their function on TORC1 activity and metabolism.

In summary, the present study reveals that appropriate levels of dietary RJ can significantly improve the survival of *nprl3*-mutant flies, which could be a rapid and cost-effective tool for the qualitative evaluation of RJ.

## MATERIALS AND METHODS

### Fly stocks and maintenance

The stocks *yw, nprl3^1^* and *Df(3L) ED4515/TM6C* (BDSC#9071) were described previously (Wei et al., 2016). The flies were maintained in an incubator at 25°C with a 12 h on/off light cycle and 60% relative humidity.

### Fly food

The standard food contained 5% cornmeal, 1% agar, 2.4% brewer's yeast, 3% sucrose, and 0.3% propionic acid, which was used as a normal diet (ND). The RJ was added to standard food at 5%, 10%, 20%, or 40% (weight/volume), and stirred during the food cooling step to make RJ food. For water-extracted RJ food, 20 g fresh RJ was dissolved in 50 ml water and centrifuged at 4000 ***g*** for 10 min. The supernatants were vacuum freeze dried, and then added to 100 ml of standard food during the food cooling step. A similar method was used to make ethanol- or ethyl-acetate-isolated RJ food. For proteinase-K-treated RJ food, the RJ was dissolved in water and the supernatant was treated with 0.1% proteinase K (Invitrogen, 25530049, USA) at 37°C for 30 min and vacuum freeze dried, and then added to the standard food at an amount that equated to 20% RJ. For high-temperature-treated RJ food, the RJ was heated at 60°C, 80°C, and 100°C for 30 min each, and added to standard food at 20% (w/v) and stirred during the food cooling step.

### The viability of *nprl3*-mutant flies

The *nprl3^1^/TM3* and *Df(3L) ED4515/TM3* (*Df*) crossed and laid eggs on standard food or RJ food for 1 day. A few days later, the number of hatched adult flies on standard food or RJ food were counted. The genotypes of flies were identified using the *Stubble* (*Sb*) marker on *TM3* balancer. While *TM3/TM3* is lethal, the expected ratio of *non-Sb* (*nprl3^1^/Df*) to *Sb* (*nprl3^1^/TM3* and *Df /TM3*) is 1:2, if the *nprl3^1^/Df* flies were fully viable. Thus, the viability ratio of *nprl3*-mutant flies is the number of the *non-TM3* flies divided by the half number of *TM3* flies*.*

### MDA, oxidase, TG, and ATP levels

The flies were crossed on standard food or RJ food. A few days later, hatched adult flies from standard food or RJ food were collected and cultured on standard food or RJ food. The 7-day-old male flies were homogenized in a buffer solution of phosphate-buffered saline (PBS) and then centrifuged for 10 min at 13,000 ***g***. The supernatant was transferred to a new tube, the MDA (A003-1, Jiancheng Bio-Engineering, Nanjing, China), SOD oxidase (A001-3, Jiancheng Bio-Engineering, Nanjing, China), CAT oxidase (A007-1, Jiancheng Bio-Engineering, Nanjing, China), triglyceride (F001-1, Jiancheng Bio-Engineering, Nanjing, China), and ATP (A095-1-1, Jiancheng Bio-Engineering, Nanjing, China) kits were used to measure the amounts of components, according to the manufacturer's instructions. The total protein was measured using the BCA Assay Kit (CW0014, CoWin Biosciences, Beijing, China) and used for normalization. The resulting data were analyzed using one-way ANOVA.

### DHE staining

The flies were crossed on standard food or RJ food. A few days later, hatched adult flies from standard food or RJ food were collected and cultured on standard food or RJ food. The abdomen from a 7-day-old male fly was dissected and incubated with 30 μM DHE (Beyotime Biotechnology, Suzhou, China) and Hoechst 33342 (H3570, Invitrogen, USA) in PBS for 5 min and then washed three times using PBS. The abdomen was fixed with 4% formaldehyde for 30 min and washed three times using PBS. The fat body was dissected from the abdomen and mounted using antifade mountant (P36982, Invitrogen, USA). Images were acquired using a confocal microscope (Zeiss LSM880, Germany). The fluorescence intensities of DHE and Hoechst, used for normalization, were quantified using Image J. The resulting data were analyzed using one-way ANOVA.

### Western blot analysis

The flies were crossed on standard food or RJ food. A few days later, hatched adult flies from standard food or RJ food were collected and cultured on standard food or RJ food for 7 days. The male flies were homogenized in 1× radioimmunoprecipitation assay (RIPA) lyisis buffer (millipore, USA) containing complete protease inhibitors and phosphatase inhibitors (Roche, USA). Immunoblot analysis was performed as described previously (Wei and Lilly, 2014). The following primary antibodies were used for western blot: pS6K (1:1000; Cell Signaling Technology, 9209, USA), total S6K (1:10,000) (Hahn et al., 2010), p4E-BP (1:500; Cell Signaling Technology, 2855, USA), total 4E-BP (1:1000; Cell Signaling Technology, 4923, USA), and β-tubulin (1:10,000; Invitrogen, MA5-16308, USA). The second antibodies were conjugated with horseradish peroxidase (HRP) (1:10,000; Jackson ImmunoResearch, USA) and visualized using an enhanced-chemiluminescence (ECL) western blot kit (CWBio, China). The relative intensities of the different bands were measured and quantified using Image J, and the resulting data were analyzed using one-way ANOVA.

## Supplementary Material

Supplementary information
