# Supplementary material for: Royal jelly attenuates metabolic defects in a Drosophila mutant with elevated TORC1 activity
Source: Biol Open. 2020 Nov 6;9(11):bio054999. doi: 10.1242/bio.054999 (PMC7657477; doi:10.1242/bio.054999)
Supplement: Supplementary information [file biolopen-9-054999-s1.pdf]

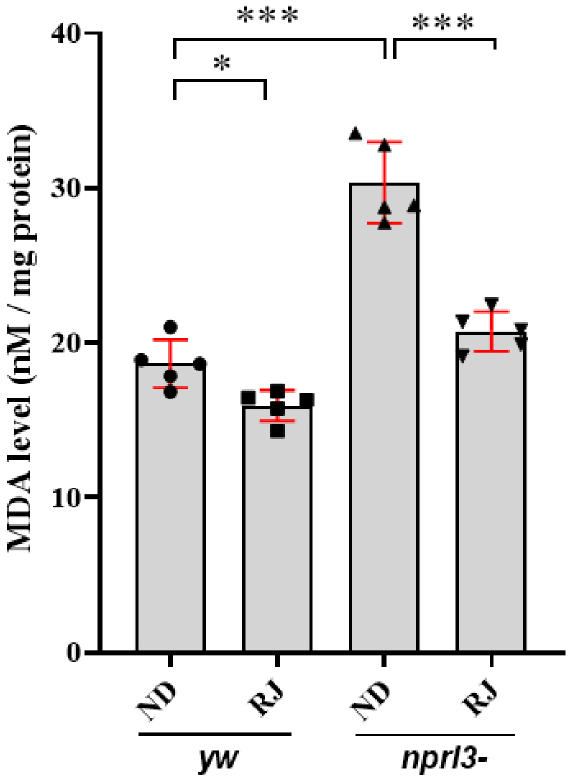

Figure S1. Effect of RJ on the reactive oxygen species levels in the *nprl3* mutant larvae. The 3rd star larvae of *yw* and *nprl3*^{1}/*Df* were collected from normal food or RJ food. The MDA levels were determined. Data are presented as mean ± SD. Values from five independent experiments. *P<0.05, **P<0.01, ***P<0.001. *yw*, control flies; *nprl3*$^{-}$, *nprl3*^{1}/*Df* flies; ND, normal diet; RJ, 20% RJ diet.

Biology Open • Supplementary information

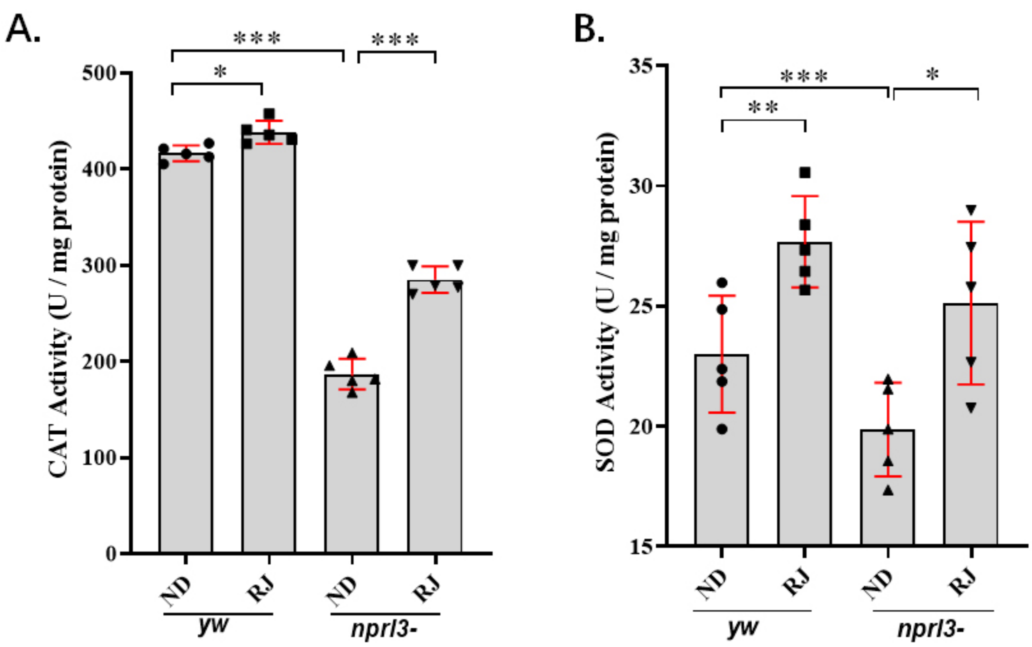

Figure S2. Effect of RJ on the antioxidase activities in the *nprl3* mutant larvae. The 3rd star larvae of *yw* and *nprl3*[1]*/Df* were collected from normal food or RJ food. **(A)** SOD activities and **(B)** CAT activities were determined. Data are presented as mean ± SD. *p<0.05, **P<0.01, ***P<0.001. Values from five independent experiments. *yw*, control flies; *nprl3*-, *nprl3*[1]*/Df* flies; ND, normal diet; RJ, 20% RJ diet.

Biology Open • Supplementary information

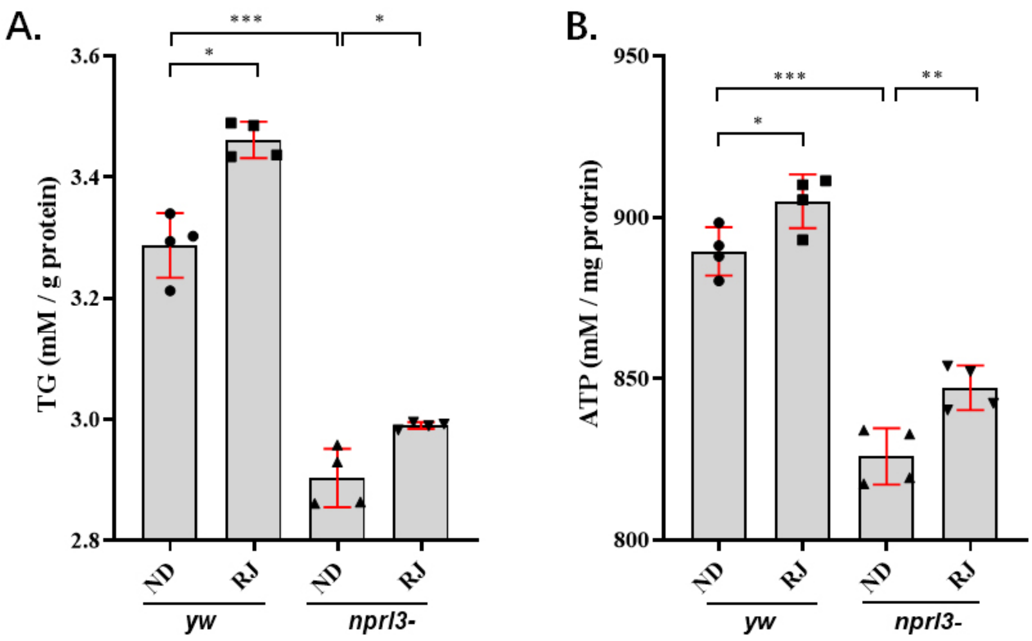

Figure S3. Effect of RJ on the metabolism in the *nprl3* mutant larvae. The 3rd star larvae of *yw* and *nprl3*[1]*/Df* were collected from normal food or RJ food. **(A)** Triglyceride (TG) levels and **(B)** ATP levels were determined. Data are presented as mean ± SD. Values from five independent experiments. *p<0.05, **P<0.01, ***P<0.001. *yw*, control flies; *nprl3*-, *nprl3*[1]*/Df* flies; ND, normal diet; RJ, 20% RJ diet.

Biology Open • Supplementary information
